# Delta Subsidence: An Imminent Threat to Coastal Populations

**DOI:** 10.1289/ehp.123-A204

**Published:** 2015-07-31

**Authors:** Charles W. Schmidt

**Affiliations:** Charles W. Schmidt, MS, an award-winning science writer from Portland, ME, has written for *Discover Magazine*, *Science*, and *Nature Medicine*.

Sea-level rise from a warming climate threatens to inundate coastlines around the world.^1^ But some of the world’s most vulnerable coasts—those fringing flat delta plains, mainly in Southeast Asia—face the far more immediate threat of sinking land.^2^ Induced mainly by human activities on a local rather than global scale, this phenomenon, known as land subsidence, can outpace sea-level rise substantially. Indonesia’s biggest city, Jakarta, is sinking at an average rate of 5–10 cm per year,^3^ much faster than the global rate of sea-level rise, which clocks in at 3.2 mm per year, according to the recent estimates.^1^ Should subsidence in Jakarta continue unabated, the city could sink up to 6 m by the end of the century, according to JanJaap Brinkman, a water management specialist with Deltares Research Institute in Delft, the Netherlands.

**Figure:**
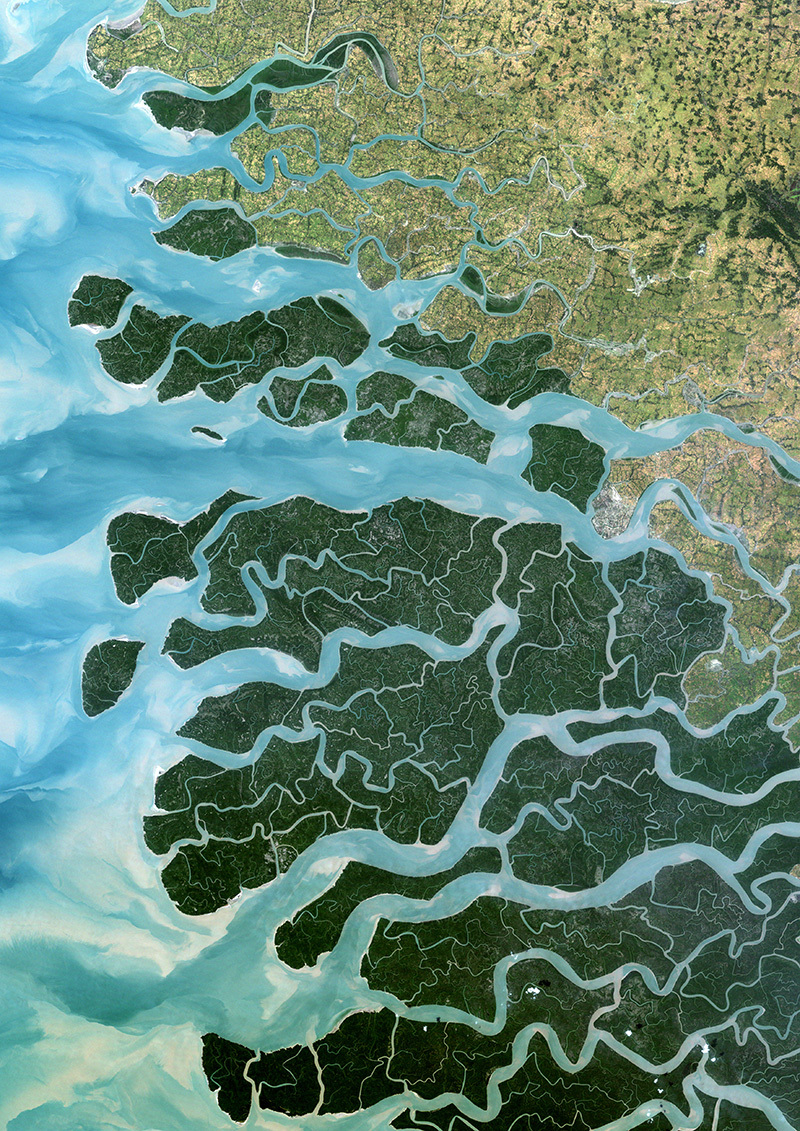
The Ganges–Brahmaputra Delta is one of several delta regions around the world that is sinking as the weight of its cities and industries combines with overextraction of natural resources from soft sedimentary deposits. © Planet Observer/Getty Images

Roughly half a billion people live in delta regions threatened by subsidence, and concerns for their well-being are mounting.^4^ For instance, in 2007 Jakarta experienced catastrophic flooding—portions of the city were inundated for weeks, 200,000 people were displaced, and roughly 1,400 were hospitalized for waterborne diarrheal diseases and by dengue fever, which is carried by mosquitoes that thrive in standing water.^5^ Marc Bierkens, a professor of hydrology at Utrecht University, the Netherlands, says Jakarta sits in a subsiding bowl where “floodwaters pool up with nowhere to go.”

Apart from the increased risk of floods and associated diseases, experts consulted for this article say subsidence threatens health in other ways. It accelerates the contamination of freshwater resources with saltwater, making them unsuitable for drinking and agriculture. Subsidence stresses gas lines, sewage pipes, and other infrastructure, which can crack as the land buckles and heaves, increasing the risk of explosions and contamination of surface and groundwater. Finally, the stress of the threat to drinking water supplies, homes, and livelihoods can adversely affect people’s sense of well-being.

Subsidence has been slowed in cities such as Tokyo^6^ and Bangkok,^7^ and the lessons learned there are galvanizing efforts to tackle the problem elsewhere. It’s also a multifaceted problem, and researchers are investigating site-specific causes of subsidence in a search for targeted solutions. But in many deltas, time is running out, warns Gilles Erkens, a senior researcher at Deltares Research Institute and Utrecht University. “In many cases, we simply don’t have ten more years to wait for more data,” he says.

## Deltaic Processes

The world’s deltas were built up mostly by aggradation, or the deposition of fertile river sediments over thousands of years.^8^ As such, they comprise important food-producing areas that attract large populations. The Mekong Delta, for instance, which is now subsiding at an average rate of 1.6 cm per year,^9^ is one of the world’s major rice exporters^10^ and home to more than 20 million people.^9^ Unlike rocky continental coasts, delta plains tend to be soft and easily compressed. They’re often propped up by underlying oil, gas, or fresh groundwater that flows through the pores of sediment deposits. As those resources are extracted, the sediments compress, and the land shrinks like a dried sponge.

Some sediments, especially those rich in organic matter, such as peat, also oxidize when they dry. Oxygen binds with carbon in the soils, creating carbon dioxide that is released to the atmosphere. Deprived of the carbon lost to this reaction, the soils lose mass and compact.^11^

**Figure:**
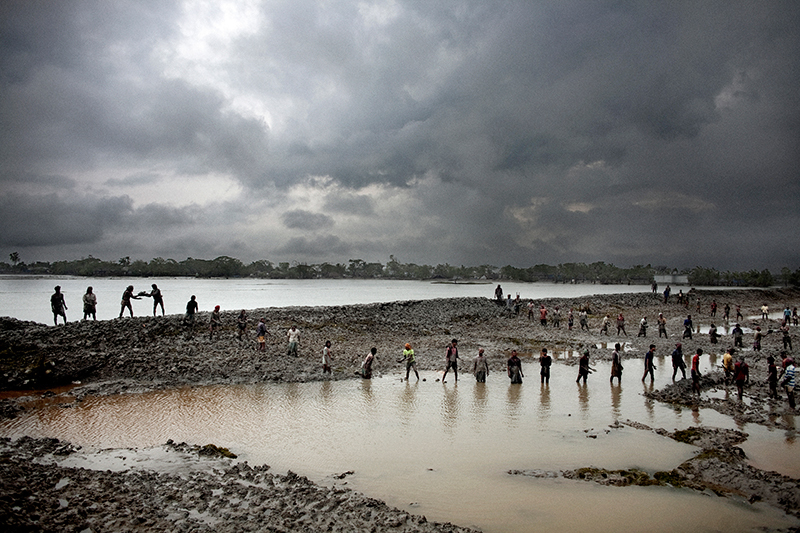
Along the Ganges–Brahmaputra Delta, earthen and concrete embankments surround low-lying agricultural areas known as polders. These embankments protect the land within, but they also block the deposition of new sediments during the rainy season. Consequently, the polders subside. When Cyclone Aila hit Bangladesh in May 2009, floodwaters crested over the embankments, turning sections of the polders to lakes. These residents are hauling mud to help build up an embankment after the cyclone, which did have one benefit—it left the area inundated with a long-overdue deposition of fresh silt, in some areas reaching 70 cm thick. © Espen Rasmussen/Panos Pictures

According to James Syvitski, an oceanographer and professor at the University of Colorado, Boulder, a delta’s elevation above sea level depends on four interrelated factors: the ocean’s global volume, aggradation, sediment compaction, and vertical movements resulting from plate tectonics and other geophysical processes. He says the ocean’s overall volume, and thus its elevation in relation to land surfaces, is increasing partly as a result of human-induced climate change. Warmer water expands, and seas are rising as vast ice sheets near the poles melt away.^1^

Aggradation has been severely limited by dams, levees, and embankments that trap silt and starve deltas of new sediments. And sediment compaction is increasing both with the extraction of groundwater and hydrocarbons, and with the growing extent of urban infrastructure. The weight of urban infrastructure compacts underlying soils, and its nonporous roofs and pavements prevent surface waters from percolating back down into the earth and recharging groundwater.

In 2009 Syvitski reported that increasing compaction and reduced aggradation had put many of the world’s deltas in danger, more than half of them in Asia. “All trends point to ever-increasing areas of deltas sinking below sea level,” he wrote. “And it remains alarming how often deltas flood, whether from land or from sea, and the trends seem to be worsening.”^4^

The biggest threat, Syvitski says, is that a delta will tip toward a collapsed state, meaning that it likely will never be restored to anything remotely similar to its natural condition. Pakistan’s Indus River Delta has already collapsed, he reported in 2013.^2^ Overexploited for agriculture, the Indus River runs dry at its discharge into the Arabian Sea nearly 140 days of the year and could soon run dry nearly year-round. The delta has shrunk to a tenth of its original size, and intruding seawater has contaminated adjacent groundwater reservoirs, submerged coastal villages, and displaced hundreds of thousands of people.

By contrast, another sinking delta in Asia—the Ganges–Brahmaputra, with a population of approximately 170 million people—hasn’t collapsed yet, but it’s getting close. Sediment delivery to the Ganges–Brahmaputra Delta has been heavily impacted by dams and levees. The worse subsidence has occurred among the jigsaw puzzle of river islands in southwest Bangladesh that together measure tens of thousands of square kilometers.^12^

To hold back the sea and create more land for agriculture, concrete and earthen embankments were built around low-lying plots of land known as polders during the 1960s. The embankments blocked replenishment of the delta with river sediment carried downstream by the annual monsoon floods, and the islands have since lost 1–1.5 m of elevation.^13^ According to Kimberly Rogers, a research associate at the University of Colorado, Boulder, they’re now far more vulnerable to storm surges that can damage or breach the walls around the polders, effectively creating lakes that can last for years. In 2009 Cyclone Aila struck southwest Bangladesh, and the resultant flooding displaced more than 100,000 people in the worst-hit areas.^14^ But the storm also inundated the islands with fresh silt, in some places reaching a depth of 70 cm,^15^ reflecting the system’s ability to replenish itself if allowed.

## Drivers of Subsidence

Scientists are improving their understanding of what drives subsidence in the Ganges–Brahmaputra Delta, yet much remains unknown. Satellite coverage over this delta was sparse until a few years ago, and there’s a chronic shortage of monitoring data.

That’s also true of Vietnam’s Mekong Delta, where Dutch and Vietnamese scientists are now collaborating on a five-year research project that could reveal opportunities to slow subsidence and limit its effects. Dubbed the Rise and Fall project, it was launched in March 2015 with $1 million in funding primarily from the Netherlands Science Founation.

**Figure:**
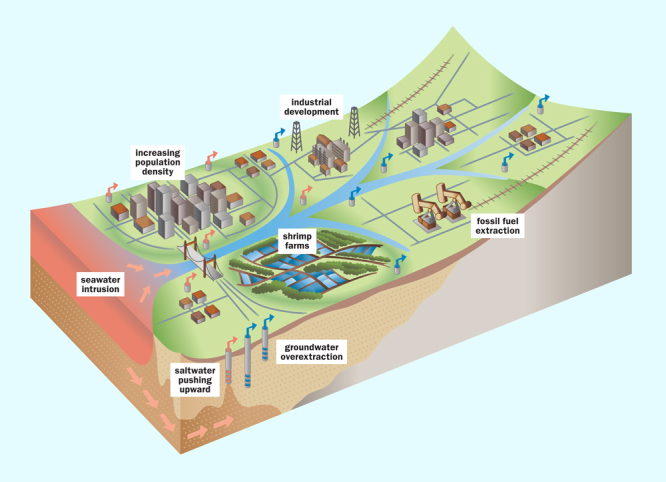
Several interconnected factors contribute to land subsidence in delta regions. Delta plains were built up from thousands of years’ worth of silt deposition, producing richly fertile lands. Populations are skyrocketing in these agricultural areas, with many serious repercussions for the land. Construction of levees, dams, and embankments blocks the natural deposition of fresh silt, depriving the land of replenishment and creating bowls where floodwaters pool with nowhere to go. Extraction of groundwater and the fossil fuels that often underlie deltas allows the land to deflate. The sheer weight of growing urban and industrial infrastructure further compresses the land, and impervious surfaces—roofs and pavements—prevent the replenishment of groundwater. Saltwater intrusion, which occurs naturally in most coastal areas, is exacerbated as the depletion of groundwater reduces water pressure. Growing populations and water-intensive industries such as shrimp farming place a heavy demand on groundwater resources. Wells must be drilled deeper, and the water coming up is saltier, as ancient seawater is pulled up by excessing pumping. © Daniel Gallant; adapted from materials provided by Deltares Research Institute

Project scientists are collecting data and sampling the subsurface geology of the Mekong Delta. They plan to create a sophisticated hydrogeological model that predicts subsidence and saltwater intrusion rates over varying scenarios of population and economic growth. “What we’re ultimately trying to do is develop more sustainable management strategies for the Mekong Delta,” says team leader Esther Stouthamer, an earth scientist and associate professor at Utrecht University.

Overexploited by domestic, industrial, and agricultural users, groundwater tables in Vietnam are falling dramatically. In the past, rice farmers relied on networks of freshwater canals for irrigation and domestic uses. These canals were the route by which new sediment was added to the land during the rainy season. But after Vietnam’s communist government opened the economy in 1986 and encouraged rice exports, villagers used their growing wealth to drill private wells, which strained groundwater resources and disrupted a centuries-old system that favored resedimentation. More than a million wells have since been drilled into the Mekong Delta, and subsidence has been accelerating ever since.^9^

**Figure:**
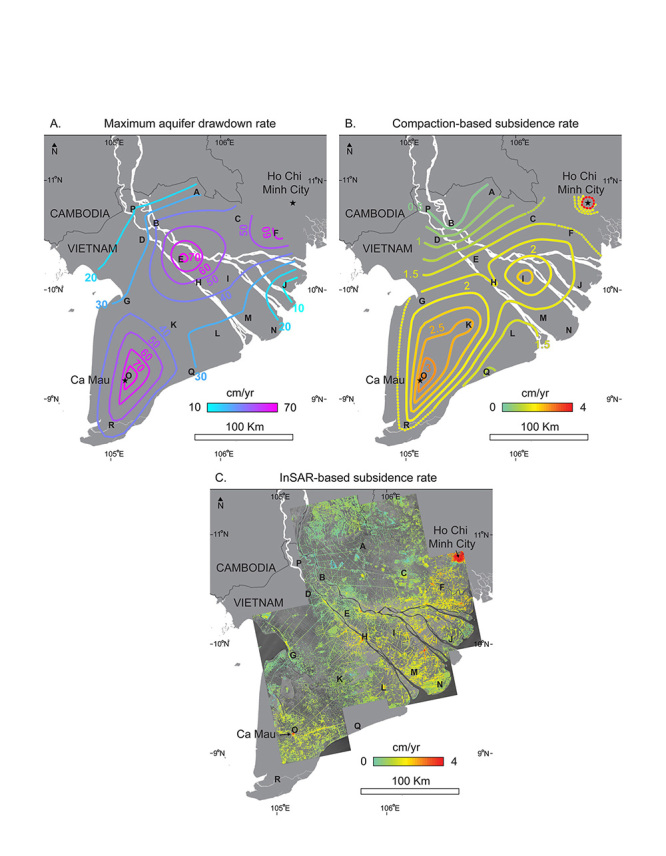
In a study of subsidence in the Mekong Delta over the period 1995–2010, Laura Erban and colleagues used well-monitoring data to estimate annual average rates of aquifer drawdown (A) and associated compaction-based subsidence at the well locations (B). These estimates corresponded closely to subsidence rates estimated from satellite imagery (C). Overall, the Mekong Delta is estimated to be subsiding at a rate of 1.6 cm per year. Source: Erban et al. (2014)^9^

Meanwhile, rice farming has given way to a more lucrative shrimp industry with an insatiable need for fresh groundwater. Laura Erban, a hydrogeologist at Stanford University, explains that shrimp are raised in brackish ponds, but their yields decline if the water gets too salty. So shrimp farmers use groundwater to continually dilute the ponds, which now stretch, one after the other, along coastlines stripped of the mangrove forests that once protected these shores.

Overexploitation is especially a problem in Cà Mau province, on the Delta’s southern tip.^9^ More than 100,000 wells have been drilled in the province, shrimp farms abound, and the urban population soared from approximately 66 million in 1990 to 90 million in 2013.^16^ Subsidence in Cà Mau now averages about 3 cm per year, according to a recent analysis by Erban.^9^

Meanwhile, groundwater pumps are drilling deeper to reach groundwater, and the water that comes up is saltier. That’s also the case along a heavily populated coastal stretch from Cà Mau to Ho Chi Minh City, about 250 km to the north.^9^ At the Rise and Fall kickoff meeting on 11 March 2015, a water official from Sóc Trăng province, located between Cà Mau and Ho Chi Minh City, reported that salt levels in groundwater in the province were climbing steadily and had reached as high as 4.2 g/L in some wells sampled in 2013. Water containing more than 2–3 g/L of total dissolved solids is generally considered too salty to drink.^17^

Gualbert Oude Essink, a hydrogeologist at Deltares Research Institute and associate professor at Utrecht University, says saltwater is penetrating farther into the Mekong Delta every year. Being heavier than freshwater, saltwater migrates down through sediments into shallow aquifers from above, he explains. That makes the groundwater increasingly nonpotable. Furthermore, he says, salt ions also react chemically with the sediments, making the ground more prone to oxidation, compaction, and therefore subsidence.

Where there’s a lot of pumping, saltwater can also contaminate fresh groundwater resources from below. Oude Essink explains that fresh groundwater typically resides over more ancient seawater that can be pulled upward by excessive pumping. That process usually takes several years. Yet it can take much longer—decades or more—for the salt levels in contaminated freshwater aquifers to decline once extraction has ceased. That’s because compared with the pumping pressure that draws saltwater up, the gravity that pulls it down is a much weaker force, Oude Essink explains.

A newer concern is that excessive pumping also could introduce arsenic into deep groundwater aquifers that would otherwise be free of the contaminant. Erban and colleagues reviewed arsenic measurements from nearly 43,000 deep wells in the Mekong Delta and found that many of them had become contaminated over time. It appears that excessive pumping could force that arsenic into deep groundwater, threatening the health of those who drink it. Erban speculates that pumping-related subsidence effectively squeezes dissolved arsenic from the clay layers as they compact. These findings contradict earlier assumptions that intervening clay layers protect deep aquifers from shallow arsenic contamination.^18^

## Tackling the Problem

Japan was one of the first countries to show that shifting away from groundwater use can slow subsidence. Subsidence was detected in Tokyo in the early twentieth century, when city officials were monitoring a pumping-induced drop in the local water table. After World War II, groundwater pumping fell off in the heavily damaged city, the water table rose, and subsidence slowed noticeably.^6^

**Figure:**
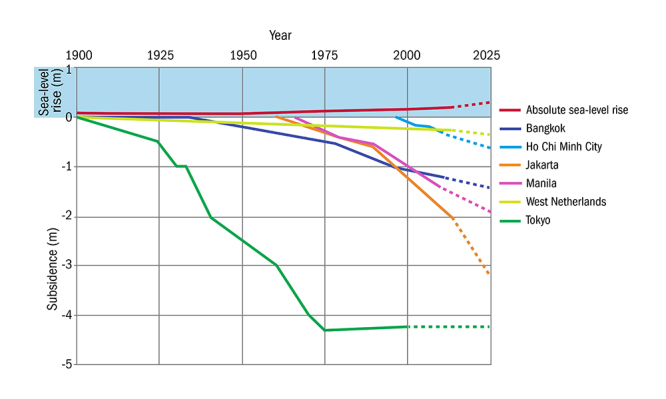
Data from Deltares compare historical subsidence rates from coastal areas around the world with estimates of absolute global sea-level rise. These are average rates; subsidence can differ considerably within a given city, depending on groundwater levels and subsurface characteristics. In some cities subsidence is accelerating as a result of economic growth. Tokyo, however, has shown that locally based mitigation measures can help stem the trend. © Erkens et al. (2015)^21^

As Japan’s economy came back to life, groundwater use picked back up, and by 1968 subsidence in some places peaked at 24 cm per year. The Tokyo Metropolitan Government imposed strict regulations on groundwater consumption, and by 2006 subsidence once again had been reduced, reaching about 1 cm per year in areas that previously had been most affected.^6^ Oude Essink points out that, while this is a dramatic reduction, it still adds up to a meter of subsidence per century.

Bangkok, Thailand, is another success story. Located in the Chao Phraya River Delta, Bangkok was subsiding by as much as 12 cm per year in the 1980s, when groundwater consumption averaged an estimated 1.2 million m^3^ per day.^7^ In 1985, with foundering infrastructure, worsening floods, and skyrocketing costs of pumping storm water into the sea, the government raised groundwater taxes sharply, according to Oranuj Lorphensri, director of the Bureau of Groundwater Control in Bangkok. She says groundwater use has since fallen to 0.8 million m^3^ per day, and subsidence has been reduced to 1–2 cm per year. To make up for groundwater declines, Lorphensri says, Bangkok shifted to using treated surface water from the nearby Chao Phraya River.

Jakarta, which Deltares’ Brinkman says currently pumps an estimated 180–250 million m^3^ of groundwater per day (including both licensed and nonlicensed groundwater uses), now faces similar prospects. According to Brinkman, the extraction of deep groundwater under Jakarta has accelerated the compaction of overlying clays. The trend is especially pronounced in northwest Jakarta, where subsidence can reach 20 cm per year.^3^ Brinkman adds that shallow groundwater contaminated with surface pollution can remain trapped under the city for weeks or months during the dry season, when there is no water to flush the low-lying northern parts of the system.

Erkens says the city’s high-rise buildings, hotels, and industry prefer deep groundwater over treated surface water because the quality of the latter is poor by comparison. “It’s a chicken-and-the-egg problem,” he says. “Companies that supply treated surface water complain they have few users and not enough money for upgrades, but then they can’t grow the customer base because the water quality is unreliable.”

But Brinkman emphasizes that Jakarta has few alternatives. If city officials cannot curtail deep groundwater use substantially within the next five years, he says, by 2030 either the population of northwest Jakarta—currently 4 million people and counting—will have to be evacuated to higher ground, or the Bay of Jakarta must be closed by a giant seawall planned by the government.^19^ “That’s the reality,” Brinkman says.

Meanwhile, officials in Vietnam remain hopeful that research will point to remedies other than limits on freely available groundwater, which is an engine for economic growth. At the Rise and Fall project’s kickoff meeting, some officials were skeptical that groundwater exploitation is what drives subsidence in the Mekong Delta. “People just say ‘groundwater is causing this,’ but we have no data to prove it,” says Bui Tran Vuong, deputy director general of the Division of Water Resources, Planning, and Investigation for South Vietnam.

Stouthamer insists accumulated evidence from around the world points to groundwater overuse as the main culprit. But she agrees that other factors are likely involved, such as the compaction that results when urban infrastructure is built on poorly supported clay or peat sediments. Changing codes so that infrastructure is engineered for better support from below and built using lighter-weight materials could help with subsidence, she says.

Another possible option is to pump water back underground to counteract subsidence. Known as managed aquifer recharge (MAR),^20^^,^^21^ this can be a controversial proposition. Syvitski warns it could have unpredictable consequences. “Even if you could do it, roads and buildings would buckle as the land rises.” Oude Essink disagrees, saying that MAR projects around the world show it to be a potentially worthy approach for reducing the groundwater declines, and therefore subsidence.

Diking huge stretches of delta shoreline would likely be problematic, as indicated by Bangladesh’s experience with polders. Dikes allow the land they protect to subside, Syvitski says, and they must be routinely elevated to keep pace with steadily rising seas.

These examples illustrate the challenges of addressing a creeping problem that’s barely perceptible to the population in real time. It’s hard to notice a drop in land elevation of a few centimeters per year until its consequences materialize in a catastrophic event, such as a devastating flood. Yet over time, these declines become significant. Where sea level is rising by an estimated 32 cm per century,^1^ land subsiding by 10 cm per year will sink that far in just over three years. Although sea-level rise gets most of the attention, for vast numbers of people worldwide, subsidence is by far the more immediate problem. But because subsidence is a local problem, local solutions are needed to keep it bay.
